# EZH2-inhibitor DZNep enhances apoptosis of renal tubular epithelial cells in presence and absence of cisplatin

**DOI:** 10.1186/s13008-020-00064-3

**Published:** 2020-05-25

**Authors:** Si-qi Chen, Jia-qi Li, Xiao-qiao Wang, Wen-jing Lei, Hao Li, Jiao Wan, Zheng Hu, Yao-wei Zou, Xiao-yu Wu, Hong-xin Niu

**Affiliations:** 1 Zhujiang Hospital, Southern Medical University, Guangzhou, 510282 China; 2 Division of Nephrology, Nanfang Hospital, Southern Medical University, North Guangzhou Ave 1838, Guangzhou, 510515 People’s Republic of China; 3 Special Medical Service Center, Zhujiang Hospital, Southern Medical University, Guangzhou, 510282 China

**Keywords:** EZH2, Apoptosis, Deptor, Renal tubular cells

## Abstract

**Background:**

The enhancer of zeste homolog 2 (EZH2) is a histone methyltransferase and induces the trimethylation of histone H3 lysine 27 (H3K27me3) in the promoter of many key genes; EZH2 acts as a transcriptional repressor and is an epigenetic regulator for several cancers. However, the role of EZH2 in nonneoplastic diseases, such as kidney diseases, is unknown and has been investigated.

**Materials and method:**

NRK-52E cells were treated with DZNep, a potent inhibitor of EZH2, with different concentrations and for different times to evaluate the apoptosis level of NRK-52E cells by Western blot and Flow cytometry analysis. The binding of EZH2 to the Deptor promoter was determined by ChIP assay.

**Results:**

The inhibition of EZH2 with 3-deazaneplanocin A (DZNep), a specific inhibitor of EZH2, led to the apoptosis of NRK-52E cells and the inhibition of mTORC1 and mTORC2 activity. A ChIP assay demonstrated that EZH2 bound the promoter region of Deptor, an endogenous inhibitor of mTORC1 and mTORC2, and regulated the transcription of Deptor by modulating H3K27me3 in its promoter region. Further experiments were performed to examine the effects of EZH2 inhibition on cisplatin-induced injured cells. Cisplatin induced the activation of mTORC1 and mTORC2 and apoptosis in NRK-52E cells, and DZNep inhibited mTORC1 and mTORC2 activity and aggravated cell apoptosis.

**Conclusions:**

These data suggested that EZH2 inhibition increased the transcription of Deptor by modifying H3K27me3 in its promoter region, subsequently inhibited mTORC1 and mTORC2 activities, downregulated the expression of apoptosis suppressor genes, and finally led to apoptosis in renal tubular cells. The inhibition of EZH2 aggravated the cisplatin-induced injury in renal tubular cells by inactivating the mTOR complexes. The present study provides new insight into renal protection and suggests that EZH2 might be a target.

## Background

The apoptosis of renal tubular cells plays an important role in the pathogenesis of kidney injury, including acute kidney injury (AKI) [1, 2]. Recent studies have found that epigenetic factors are involved in signal transduction and information transmission in the initiation and progression of renal tubular cell apoptosis [3, 4].

The enhancer of zeste homolog 2 (EZH2) is a catalytic subunit of the polycomb repressive complex 2 (PRC2), acts as a histone methyltransferase and induces the trimethylation of histone H3 lysine 27 (H3K27me3) in the promoter of many key genes. EZH2 acts as a transcriptional repressor and epigenetic regulator, allowing it to regulate gene expression [5]. EZH2 was initially detected in tumors, such as breast cancer, colorectal cancer, prostate cancer [6]. EZH2 can inhibit the expression of several key tumor suppressor genes by its histone methylation function and accelerate the poor prognosis of tumors [5, 7, 8]; thus, EZH2 is regarded as a reliable biomarker for tumor progression and a potential parameter in the degree of malignancy and prediction of poor outcome [9, 10]. Despite its crucial role in tumors, EZH2 has a role in dental pulp inflammation [11] and prostatitis [12] and has attracted attention for its function in nonneoplastic diseases. However, even in the few studies, the role of EZH2 in kidney diseases remains largely controversial. For example, Zhou’s study shows that in a renal fibrosis model, EZH2 depletion alleviates renal fibrosis [13]. Consistently, Wan’s research demonstrates that a decrease of EZH2 is beneficial for podocyte injury by antagonizing Wilm’s tumor 1 (WT1) in diabetic nephropathy [14]. However, another study presents the opposite view. Siddiqi et al. [15] observed that in rats with diabetic nephropathy, EZH2 depletion promotes oxidative stress and programmed cell death in podocytes. Based on the above analysis, we wonder, what roles does EZH2 actually play and what is the function of EZH2 in the apoptosis of renal tubular epithelial cells?

In cancer cells, EZH2 epigenetically represses Deptor, an inhibitor of the mammalian target of rapamycin (mTOR) pathway [16]. mTOR is a serine/threonine (Ser/Thr) protein kinase that primarily functions as a vital regulator of cell proliferation, growth, and survival. mTOR interacts with several proteins including Deptor to form two distinct complexes named mTOR complex 1 (mTORC1) and 2 (mTORC2) [17–20]. Deptor interacts with a C-terminal portion of mTOR, which is upstream of its kinase domain, through the PDZ domain and acts as a negative regulator of mTORC1 and mTORC2 [19, 21]. Thus, the regulation of Deptor expression by EZH2 may control cell growth and proliferation through mTOR complex pathways.

In the present study, we identified that the inhibition of EZH2 with 3-deazaneplanocin A (DZNep) upregulated the transcription of Deptor by decreasing the H3K27me3 methylation level in its promoter region and reduced the activity of mTORC1 and mTORC2, resulting in apoptosis of NRK-52E cells. Moreover, due to the inhibition of mTOR complex activity by EZH2 inhibition, cisplatin-induced apoptosis was decreased in NRK-52E cells. These data provide new insight into the pathogenesis of apoptosis in renal tubular cells and suggest that EZH2 might be a potential target for protecting kidney injury, such as AKI.

## Results

### Inhibition of EZH2 induced apoptosis in NRK-52E cells

**Fig. 1:**
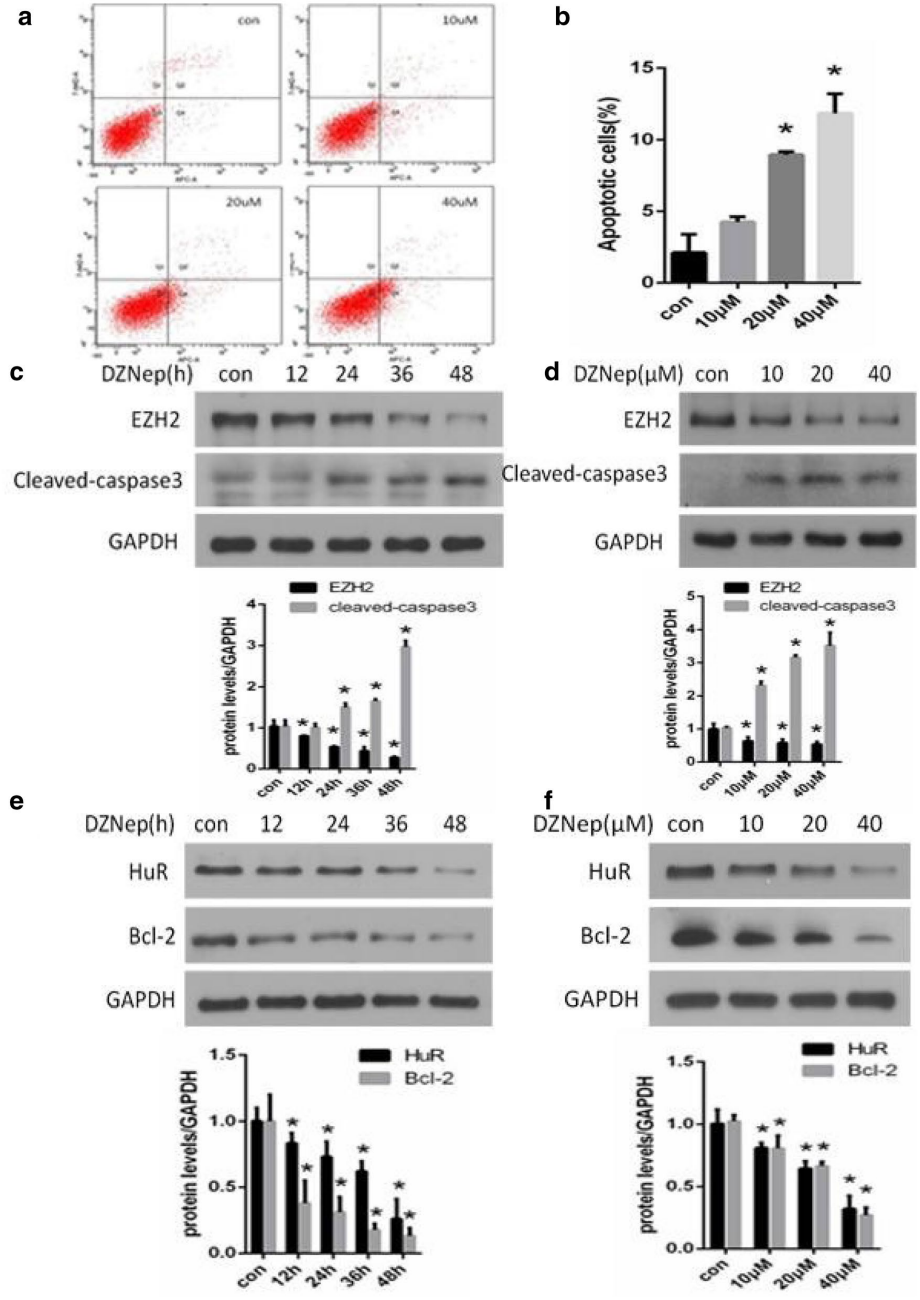
EZH2 inhibition induced apoptosis in NRK-52E cells and inhibited of HuR expression. NRK-52E cells were stimulated with 0, 10, 20, and 40 μM DZNep (3-deazaneplanocin A) for 24 h. Flow cytometry analysis revealed obvious apoptosis after DZNep treatment. Quantitative analysis of cell apoptosis was performed by flow cytometry, and apoptotic cells were identified as Annexin V^+^7-AAD^−^ (**a**). NRK-52E cells were treated with various concentrations of DZNep for 24 h and the percentage of apoptotic cells was measured (**b**). NRK-52E cells were stimulated with 20 μM DZNep for the indicated times (**c**, **d**) or stimulated with different concentrations of DZNep for 24 h (**e**, **f**). Western blots showed the protein level of EZH2, cleaved-caspase 3, HuR and Bcl-2; GAPDH was used to verify equivalent loading. Quantitative data are presented. Data are given as the mean ± SD of three independent experiments. *p < 0.05 versus vehicle-treated cells

NRK-52E cells were treated with different concentrations of DZNep, a potent inhibitor of EZH2, for the indicated time. As shown in Fig. 1c, the protein level of EZH2 was decreased in a time- and dose-dependent manner by western blot, indicating that DZNep inhibited EZH2 expression. Accordingly, obvious apoptosis in NRK-52E cells was revealed after DZNep treatment as detected by flow cytometry (Fig. 1a). In addition, the apoptosis of NRK-52E cells was dose-dependent with the concentration of DZNep (Fig. 1b). Consistently, cleaved caspase 3, an apoptosis-related protein, was significantly increased in a time- (Fig. 1c) and dose-dependent (Fig. 1d) manner.

On the other hand, the expression of Bcl-2, an anti-apoptosis gene, showed convincing downregulation in a time- (Fig. 1e) and dose-dependent (Fig. 1f) manner after DZNep treatment. Previous studies demonstrated that Human antigen R (HuR) enhances the expression of anti-apoptotic genes by binding anti-apoptotic mRNAs and encoding anti- apoptotic proteins. Bcl-2 mRNA was recently identified as a HuR target [22]. Therefore, we investigated whether EZH2 inhibition influenced the expression of HuR. Western blot analysis demonstrated that HuR protein was also down-regulated in a time- (Fig. 1e) and dose-dependent (Fig. 1f) manner.

These data suggested that EZH2 inhibition induced notable apoptosis in NRK-52E cells.

### Inhibition of EZH2 decreased the activity of mTORC1 and mTORC2

**Fig. 2:**
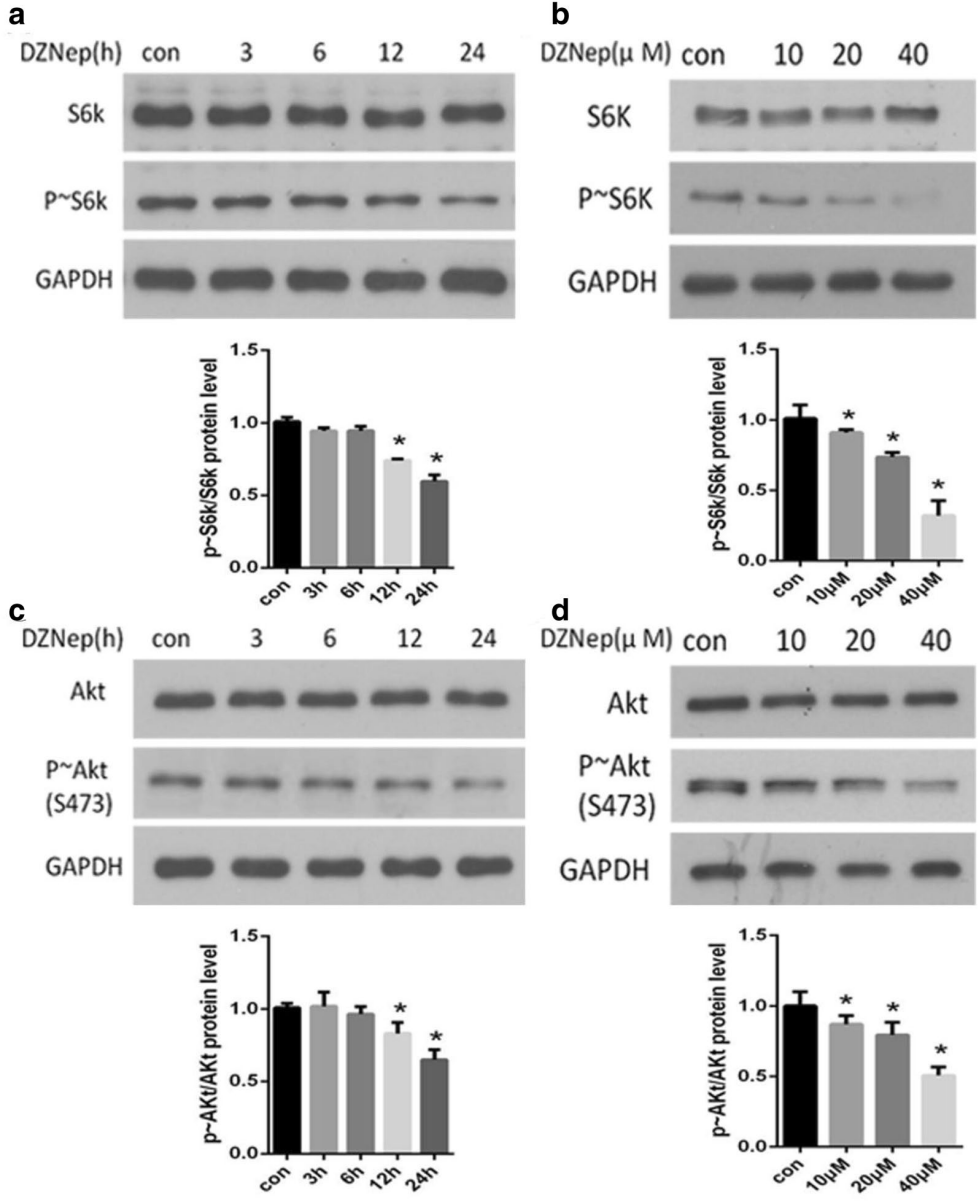
EZH2 inhibition repressed mTORC1 and mTORC2 activity. NRK-52E cells were stimulated with 20 μM DZNep for the indicated times (**a**, **c**) or stimulated with different concentrations of DZNep for 24 h (**b**, **d**). Western blots were used to measure the protein level of p ~ S6K, S6k, p ~ Akt and Akt. GAPDH was used to verify equivalent loading, and quantitative data are presented. Data are given as the mean ± SD of three independent experiments. *p < 0.05 versus vehicle-treated cells

The activities of mTORC1 and mTORC2 were evaluated by the phosphorylation levels of S6k [23] and Akt [17], respectively [19]. The protein level of p ~ S6K, as determined by western blot, decreased after exposure of NRK-52E cells to DZNep in a time- (Fig. 2a) and dose-dependent (Fig. 2b) manner. Compared with the baseline, the expression of p ~ S6K decreased at 12 h and peaked at 24 h after treatment with 20 μM DZNep (Fig. 2a). The expression of the p ~ S6K protein decreased as the concentration of DZNep increased (Fig. 2b). The total S6K protein expression was not altered (Fig. 2a, b). The same changes were observed for p ~ Akt and total Akt (Fig. 2c, d).

These data indicated that EZH2 inhibition decreased the activity of mTORC1 and mTORC2 activity.

### EZH2 regulated mTORC1 and mTORC2 activity through epigenetic regulation of deptor

**Fig. 3:**
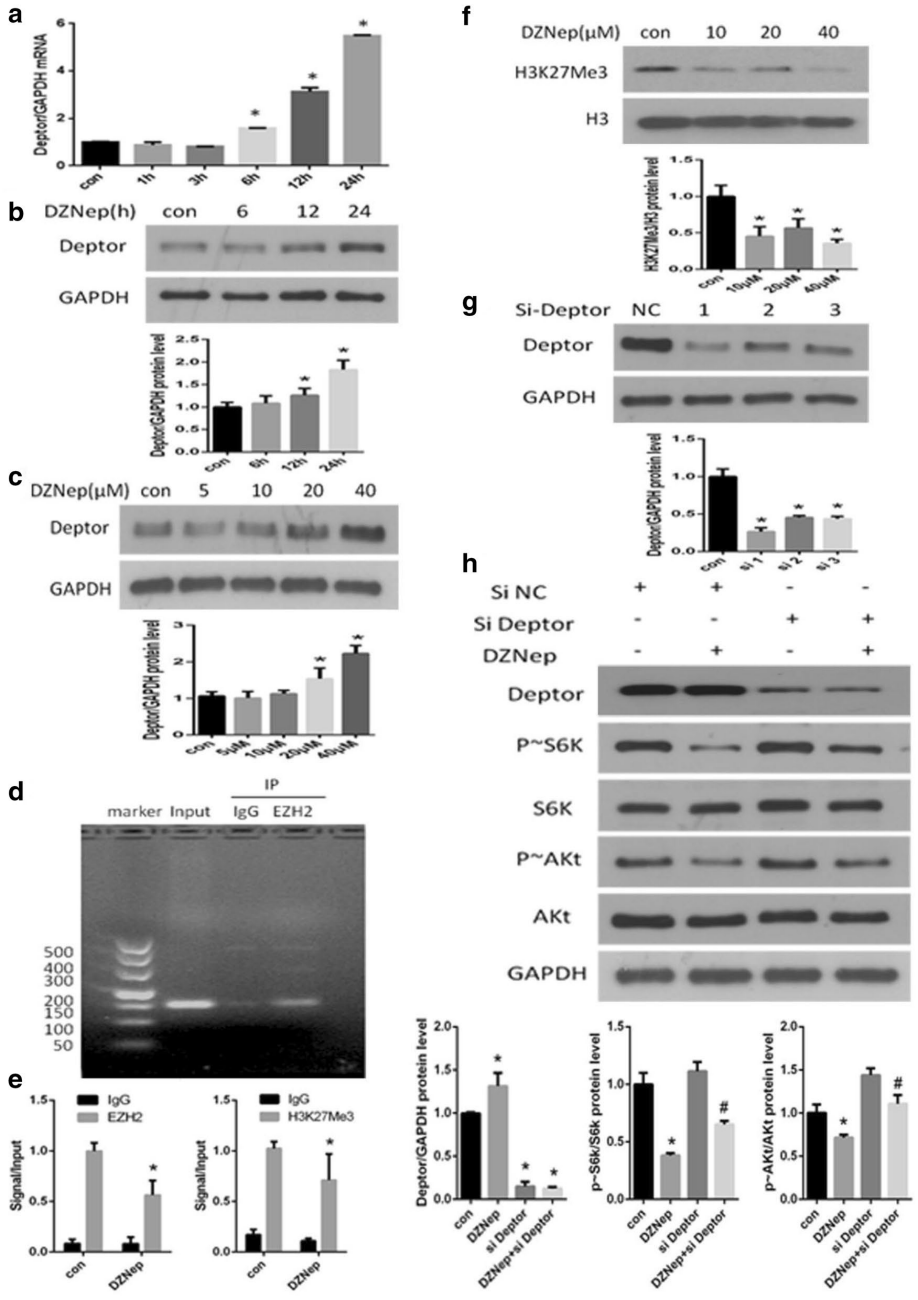
EZH2 epigenetically regulated the expression of Deptor to affect mTORC1 and mTORC2 activity. NRK-52E cells were treated with 20 μM DZNep for various times. The mRNA and protein levels of Deptor were measured by RT-qPCR (**a**) and western blot (**b**), then, we treat NAK-52E cells with different concentrations of DZNep for 24 h, the protein levels of Deptor were measured by western blot (**c**). The promoter of Deptor was ChIP-ed with an anti-EZH2 antibody or IgG control (**d**). The promoter of Deptor was ChIP-ed with an anti-EZH2 or anti-H3K27me3 antibody or IgG control in cells with or without DZNep stimulation. In the second bar, the p-value of the IgG decrease was greater than 0.05, which was not statistically significant (**e**). NRK-52E cells were treated with various concentrations of DZNep for 24 h. The protein level of H3K27me3 was measured, and H3 was used to verify equivalent loading (**f**). NRK-52E cells were transfected with three different siDeptor or negative control siRNAs for 24 h, and the protein level of Deptor was measured (**g**). NRK-52E cells were transfected with Deptor siRNA for 24 h, followed with 20 μM DZNep stimulation. After 24 h, the protein levels of Deptor, p ~ S6k and p ~ Akt were measured (**h**). Data are given as the mean ± SD of three independent experiments. *p < 0.05 versus vehicle-treated cells. # p < 0.05 versus cells treated with only DZNep

To further investigate the mechanisms underlying the inhibition of mTORC1 and mTORC2 activity by EZH2 inhibition, we examined Deptor. Deptor is a recently identified inhibitor of the mTOR kinase. Both mTORC1 and mTORC2 are directly inhibited by Deptor, which binds mTOR through its PDZ domain [19]. We found that EZH2 inhibition accompanied an increase in Deptor transcription level. DZNep treatment upregulated Deptor expression at both the mRNA (Fig. 3a) and protein levels (Fig. 3b, c) in NRK-52E cells.

EZH2 epigenetically represses several negative regulators of the mTOR pathway in tumors, including Deptor [16]. Here, we performed a chromatin immunoprecipitation (ChIP) assay to verify whether this regulation also exists in NRK-52E cells. EZH2 bound the Deptor promoter region and then regulated its transcriptional level (Fig. 3d). When treated with DZNep, the protein levels of both EZH2 and H3K27me3, which are found at the promoter region of Deptor, were decreased (Fig. 3e). In addition, the methylation level of H3K27 in the entire cell was dose-dependently downregulated in the presence of DZNep (Fig. 3f).

To further validate the relationship between Deptor regulation and mTORC1 and mTORC2 activity, we used siRNA to inhibit the expression of Deptor in NRK-52E cells and tested the effectiveness of Deptor siRNA (Fig. 3g). We chose the most effective sequence and used the selected siRNA sequence to transfect NRK-52E cells. After treatment with DZNep for 24 h, the protein levels of p ~ S6K and p ~ Akt were tested. As shown in Fig. 3h, the knockdown of Deptor with siRNA apparently rescued the inhibition of mTORC1 and mTORC2 activity induced by EZH2 inhibition.

These data indicated that EZH2 inhibition decreased mTORC1 and mTORC2 activity by up-regulating Deptor expression.

### Inhibition of EZH2 enhanced cisplatin-induced injury in NRK-52E cells

**Fig. 4:**
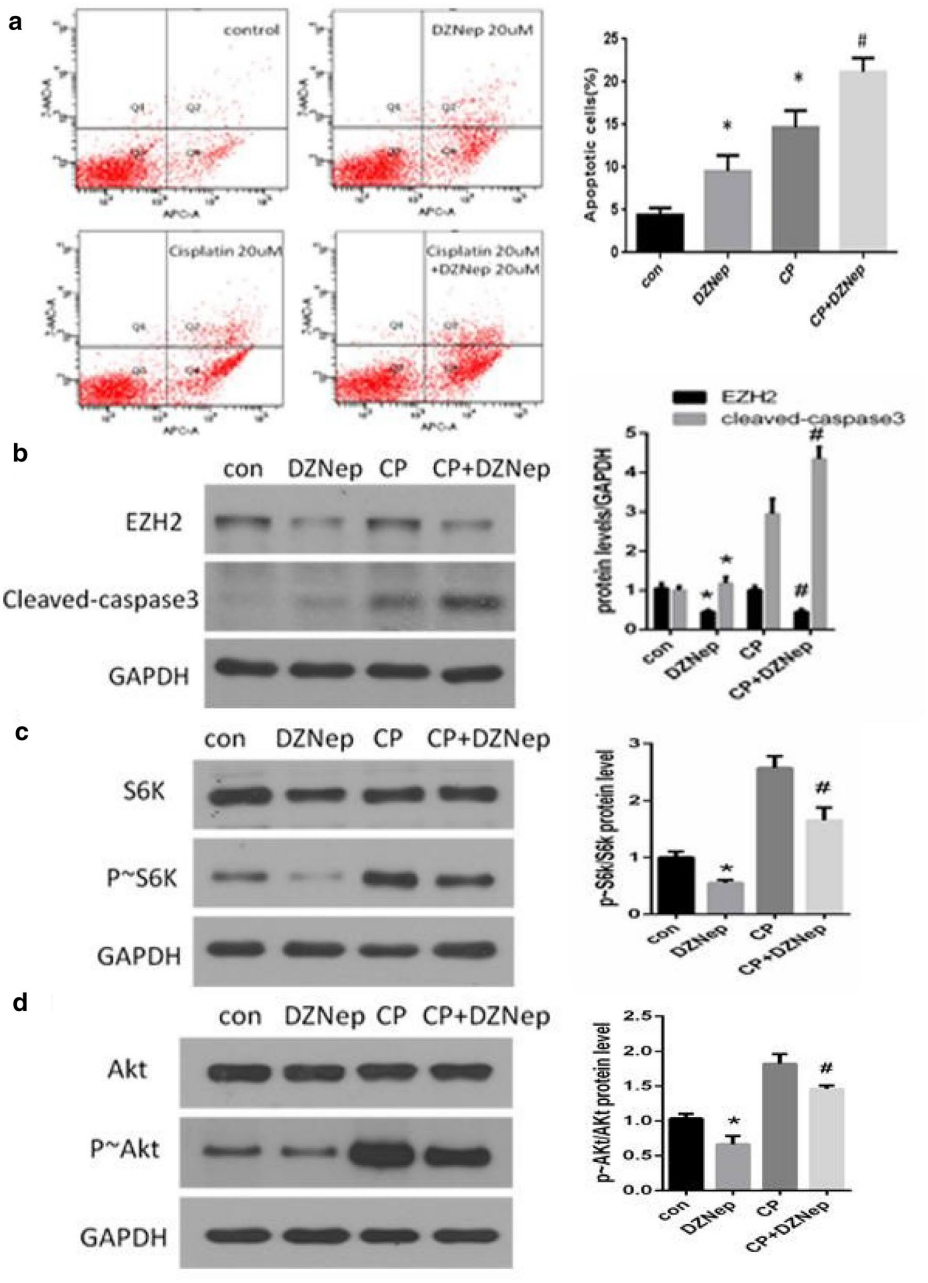
EZH2 inhibition aggravated cisplatin-induced apoptosis in NRK-52E cells. NRK- 52E cells were stimulated with 20 μM DZNep for 1 h, followed by 20 μM cisplatin (CP) stimulation for 24 h. Flow cytometry analysis was conducted (**a**). Western blots show the protein level of cleaved-caspase 3 (**b**), S6k, p ~ S6k (**c**), Akt and p ~ Akt (**d**). GAPDH was used to verify equivalent loading. Quantitative data are presented. Data are given as the mean ± SD of three independent experiments. *p < 0.05 versus vehicle- treated cells. #p < 0.05 versus cells treated with cisplatin alone

To examine the effects of EZH2 inhibition on cells that were injured by cisplatin, NRK-52E cells were preincubated with 20 μM DZNep for 1 h and subsequently treated with 20 μM cisplatin for 24 h. As shown by flow cytometry, cisplatin induced apoptosis in NRK-52E cells. The apoptosis level in NRK-52E cells preincubated with DZNep was further increased compared with that of cisplatin stimulation alone (Fig. 4a). In agreement with the apoptosis rate, the protein level of cleaved-caspase 3 also markedly increased in cells preincubated with DZNep compared with that of cisplatin stimulation alone. Cisplatin had no effect on EZH2 expression (Fig. 4b).

As shown in Fig. 4c, d, the phosphorylation level of S6K (p- S6K) and Akt (p-Akt) was increased after cisplatin treatment. DZNep preincubation led to a significant downregulation of p ~ S6K (Fig. 4c) and p ~ Akt (Fig. 4d) protein expression.

These data suggested that EZH2 inhibition aggravated apoptosis in NRK-52E cells injured by cisplatin by decreasing mTORC1 and mTORC2 activity.

## Discussion

EZH2 is a widely expressed and highly conserved protein that belongs to the polycomb group of genes (PcG) and plays an important role in epigenetic regulation [24]. EZH2 mainly functions as a histone methyltransferase (HMT) and acts as a transcriptional repressor by inducing the trimethylation of histone H3 lysine 27 (H3K27me3) on the promoter region of indicated genes, thus regulating cellular biological behavior [9]. The present study showed that the inhibition of EZH2 induced apparent apoptosis in cultured NRK-52E cells, as demonstrated by flow cytometry and the concomitant increase of a pro-apoptosis protein (cleaved-caspase 3) and decrease of anti-apoptosis proteins (Bcl-2 and HuR).

HuR promotes cell survival by maintaining the enhanced expression of anti-apoptotic genes at the transcriptional level, and Bcl-2 is one of the two recently identified HuR- targeted mRNAs that encodes an anti-apoptotic protein [22]. Previous studies have confirmed that activated mTORC1 promotes cell growth and protein synthesis mainly through the phosphorylation of the ribosomal S6 kinase (S6K) [23], and activated mTORC2 modulates cell survival as characterized by the phosphorylation of the Akt kinase [17]. mTORC1 and mTORC2 activated HuR through phosphorylation of HSF1 [25, 26]. In the present study, we observed that EZH2 inhibition induced a decrease in mTORC1 and mTORC2 activities as well. These data indicated that HuR is situated between the mTOR complexes and Bcl-2 and that EZH2 inhibition might inactivate mTORC1 and mTORC2 in some manner, thus downregulating HuR and Bcl-2 expression and leading to cell apoptosis.

To determine whether EZH2 functions with mTOR complexes through epigenetic regulation, we tested whether EZH2 inhibition with DZNep could inhibit histone H3K27 trimethylation, a characteristic function of EZH2. As expected, H3K27me3 expression was largely decreased in a dose-dependent manner after DZNep treatment in NRK-52E cells.

In this experiment, we demonstrated that EZH2 inhibition caused an apparent increase in Deptor expression by RT-qPCR and Western blotting. Moreover, a ChIP assay further verified the presence of binding sites for EZH2 in the promoter of Deptor. Finally, we tested the relationship between Deptor and mTOR. The upregulation of Deptor was related to the inactivation of mTOR and mTOR complexes. After inhibiting Deptor expression with siRNA, the effect of EZH2 inhibition-induced inactivation of mTOR complexes was reversed. These data indicated that EZH2 regulates mTOR complex activity through the epigenetic regulation of Deptor in renal tubular cells. This finding was consistent with the work of Wei et al. [16] who demonstrated that in tumor cells, EZH2 can be recruited to the promoter of several negative regulators of mTOR, including Deptor, and regulate their transcription. Notably, the effect of EZH2 inhibition-induced inactivation of mTOR complexes was not completely reversed after Deptor depletion, suggesting that Deptor might not be the only target of EZH2 epigenetic regulation.

These data revealed an apoptosis-related signal cascade involving mTOR complex- activated HuR and Bcl-2 in renal tubular cells. Deptor might be epigenetically regulated by EZH2 upstream of this cascade.

Activating the mTOR signal pathway by pharmacological methods can reduce the number of apoptosis-positive tubular cells in a renal ischemia/reperfusion(I/R) injury model [15], highlighting what occurs in damaged cells. The platinating agent cisplatin is commonly used in solid tumor therapy. The anticancer efficacy of cisplatin largely depends on the formation of bivalent DNA intra-strand crosslinks, which stimulate the DNA damage response, thereby triggering checkpoint activation, gene expression and cell death. The clinically most relevant adverse effect associated with cisplatin treatment is nephrotoxicity that results from damage to renal tubular epithelial cells [27]. In our study, cisplatin treatment induced apoptosis but had no effect on EZH2 in NRK-52E cells, indicating that the effects of cisplatin on apoptosis were independent on EZH2. Interestingly, there was a marked induction of S6K and Akt phosphorylation after cisplatin treatment, which was similar to that in cochleovestibular hair cells and LLC-PK1 (a pig renal tubular epithelial cell line) reported by Nicholas BD [28] and Kaushal GP [29], respectively. mTOR blocks apoptosis and promotes cell survival. While inducing apoptosis, cisplatin activates mTOR, inferring that the apoptotic pathway is a highly regulated process. Cells acquire information in response to a death stimulus to initiate the apoptotic pathway. Once initiated by an apoptotic stimulus, the cell death pathway can be challenged by cell survival signals to overcome injury and maintain cell viability. Thus, the extent of cell injury caused by a toxic agent will depend on the balance between the activation of apoptotic signals and the induction of survival signals [29]. The inhibition of EZH2 with DZNep blocked the phosphorylation of mTORC1 and mTORC2 and aggravated cisplatin-induced apoptosis. These data suggested that the inhibition of EZH2 enhanced the cisplatin-induced injury via inactivation of the mTOR complexes.

## Conclusion

**Fig. 5:**
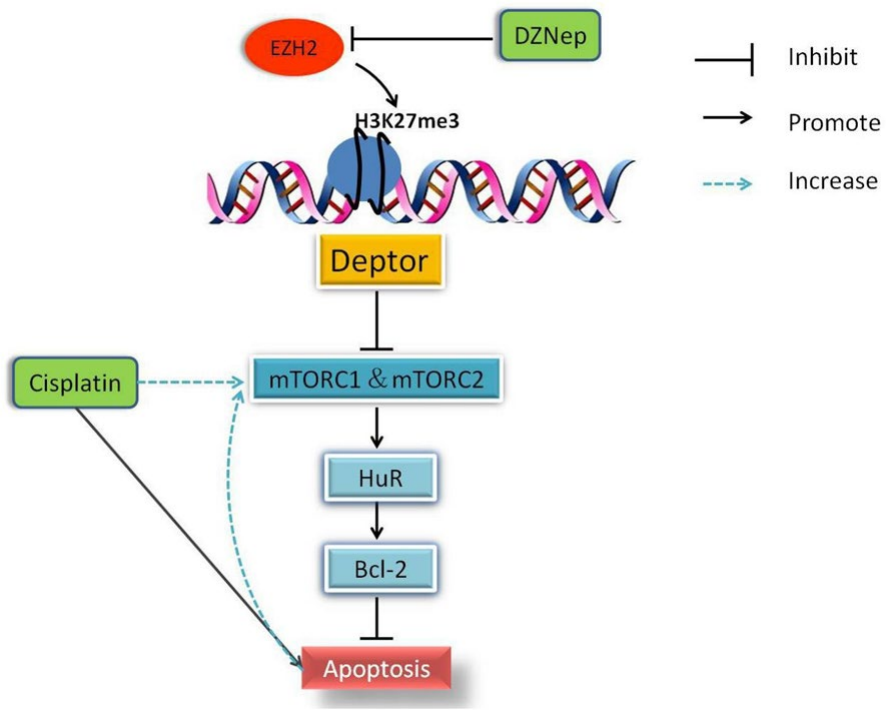
A schematic summary of the mechanisms of EZH2 inhibition on apoptosis induction in NRK-52E cells

In summary, our results showed that EZH2 inhibition increased the transcription level of Deptor by decreasing the level of trimethylation of H3K27 in the Deptor promoter region, subsequently inhibited the activities of mTORC1 and mTORC2, downregulated the expression of HuR and Bcl-2, and finally led to apoptosis in renal tubular cells (Fig. 5). The inhibition of EZH2 aggravated cisplatin-induced injury in renal tubular cells by inactivating mTOR complexes. EZH2 is indispensable for preventing apoptosis in renal tubular epithelial cells. Maintaining a moderate level of EZH2 may be helpful to protect against renal injury.

## Materials and methods

### Cell culture and reagents

NRK-52E cells (ATCC, Manassas, VA) were cultured in Dulbecco’s modified Eagle’s medium (DMEM) with 10% fetal bovine serum (Gibco/Life Technologies, NY) at 37 °C in a humidified atmosphere containing 5% CO_2_. NRK-52E cells were seeded at 4 × 10^4^ cells/ml. When the cells reached approximately 70% confluence, they were treated with various concentrations of DZNep (s7120,Selleck, China) for 24 h or 20 μM DZNep for the mentioned times at 37 °C to determine the role of EZH2 in normal cells. To observe the influence of EZH2 inhibition on cells treated simultaneously with of cisplatin, cells were assigned to four treatment groups as follows: Control, 20 μM DZNep; 20 μM cisplatin (P4394, Sigma, America); 20 μM DZNep + 20 μM cisplatin. DZNep was added 1 h before cisplatin.

### Quantitative real-time PCR

Total RNA was harvested from NRK-52E cells with Trizol reagent (Invitrogen), and cDNA was synthesized using the PrimeScript RT reagent kit from Takara. Real-time PCR was performed using the ChamQ SYBR qPCR Master Mix from Vazyme on an ABI PRISM 7500 Fast sequence detection system (Applied Biosystems, Foster City, CA). Primers used in this study were as follows: rat Deptor Forward 5′-GAGAGCAGCTCCGACTGATG-3′; Reverse 5′- TCGTTGTGGCTTCTCCTTCC-3′; rat GAPDH Forward 5′- CCATCAACGACCCCTTCATT-3′; Reverse 5′- CACGACATACTCAGCACCAGC-3′.

### Western blotting

The proteins were extracted from NRK-52E cells using PLC lysis buffer containing a cocktail inhibitor (Merck Millipore, GER). The cell lysate was subjected to western blot as described previously [30]. The primary antibodies used are as follows: anti-EZH2 (5246 s; Cell Signaling Technology), anti-Bcl-2 (3498 s; Cell Signaling Technology), anti-cleaved caspase 3 (9664 s; Cell Signaling Technology), anti-Akt (4685 s; Cell Signaling Technology), anti-p ~ Akt (4060 s; Cell Signaling Technology), anti-S6K (2708 s; Cell Signaling Technology), anti-p ~ S6K (9234 s; Cell Signaling Technology), anti-HuR (ab200342; Abcam), anti-Deptor (11816 s; Cell Signaling Technology), anti-H3K27me3 (9733 s; Cell Signaling Technology), H3 (4499 s; Cell Signaling Technology), and anti-GAPDH (5174 s; Cell Signaling Technology).

### Flow cytometry analysis

The apoptosis rate in NRK-52E cells was detected with an Annexin V-APC/7-ADD apoptosis detection kit (Becton–Dickinson, USA) according to the manufacturer’s instructions. The apoptosis level of stained NRK-52E cells was assessed by FACSCanto II Flow cytometry (Becton–Dickinson, CA, USA). Cells that were positive for Annexin V but negative for PI were defined as apoptotic cells.

### Chromatin immunoprecipitation (ChIP)

The chromatin immunoprecipitation (ChIP) assay was performed as described previously [31]. Briefly, the collected NRK-52E cells were fixed with 1% formaldehyde for 10 min at 37 °C, washed three times with cold PBS and lysed for 10 min on ice. The cross-linked samples were then subjected to sonication, yielding DNA fragments with a size of 200–1000 bp. After centrifugation at 12,000 rpm for 10 min, the soluble chromatin was incubated with anti-EZH2 (diluted 1:100) or anti-H3K27me3 (diluted 1:50) antibody or IgG. The incubation lasted overnight at 4 °C with mild rotation, and then the complexes were isolated using protein A-agarose beads and washed with low-salt, high-salt, LiCl, and Tris- EDTA buffers sequentially. Then, the complexes were extracted with freshly prepared 1% SDS-0.1 M NaHCO3 at 65 °C for 6 h, and the DNA was purified with a Qiagen DNA extraction kit. Finally, qPCR was performed, and the primers used in this study were as follows: rat Deptor Forward 5′-TCACAGACACAAGTCTCCGTATC-3′, Reverse 5′-AGCCCGAGCGTTCATTAAAAG-3′.

### RNA interference

Small interfering RNAs (siRNAs) against Deptor and the control siRNA were obtained from GuangZhou RiboBio (GuangZhou, China). siRNA knockdown was performed in accordance with the manufacturer’s protocol. Briefly, 4  ×  10^5^ cells were plated in 6-well plates for 24 h and transfected with 50 nM Deptor siRNA or control siRNA using Lipofectamine 2000 (Invitrogen, Shanghai, China) in DMEM medium. After 24 h, the extent of knockdown was analyzed by western blot. Wild-type and Deptor knockdown NRK-52E cells were stimulated with 20 μM DZNep for 24 h and cultured in DMEM medium at 37 °C in a humidified 5% CO_2_ atmosphere. The RNA sequence of Deptor RNAi oligonucleotide was ACCCATTTGTGGACAGCAA.

### Statistical analysis

All experiments were performed at least 3 times independently. The statistical analysis was performed using SPSS statistical software package SPSS 20.0 (SPSS Inc, Chicago, USA). The results are expressed as the mean ± SD, and differences between groups were determined using a one-way ANOVA or *t* test. p values of < 0.05 were considered statistically significant.

## Data Availability

The datasets used and/or analyzed during the current study are available from the corresponding authors on reasonable request.

## Abbreviations

EZH2: Enhancer of zeste homolog 2
Deptor: DEP domain-containing mTOR-interacting protein
mTORC1: Mammalian target of rapamycin complex 1
mTORC2: Mammalian target of rapamycin complex 2
HSF1: Heat shock factor 1
HuR: Human antigen R
DZNep: 3-Deazaneplanocin A
CP: Cisplatin
NRK-52E: Normal rat kidney proximal cell
